# Fixation of Metacarpal Fractures Using Intramedullary Headless Compression Screws: A Tertiary Care Institution Experience

**DOI:** 10.7759/cureus.4466

**Published:** 2019-04-16

**Authors:** Adeel A Siddiqui, Jagdesh Kumar, Muhammad Jamil, Mariyam Adeel, Ghulam M Kaimkhani

**Affiliations:** 1 Orthopaedic Surgery, Dow University of Health Sciences, Karachi, PAK; 2 Orthopedic Surgery, Civil Hospital, Karachi, PAK; 3 Orthopaedics, Dow University of Health Sciences, Karachi, PAK; 4 Orthopedic Surgery, Dow University of Health Sciences, Karachi, PAK

**Keywords:** metacarpal fractures, intramedullary headless screw, hand fractures

## Abstract

Introduction

Metacarpal fractures account for the majority of hand fractures. Inadequate management can cause functional deficit which can lead to loss of fine hand movements. Adequate management has proven to give good outcomes. The use of intramedullary screws has given better results than the use of Kirschner wires (K-wires).

Method

This study was conducted at Dr. Ruth Phau Civil Hospital, Karachi (CHK) between August 1, 2018 and January 31, 2019. A total of 32 patients presented with metacarpal fractures. They were surgically managed with intramedullary headless screw fixation. Post-operatively, grip strength, range of motion, and presence of any disabilities were recorded. Patients were followed up to three months.

Results

Out of 32 patients, six were females. Mean age was found to be 29.1 ± 10.5 years. Post-operatively the mean grip strength was found to be 37.8 ± 7.3 kilograms. The mean total active range of motion was found to be 242.8 ±14.5 degrees. The mean days to return back to work were 25 ± 5.4 days. The mean patient satisfaction score was 8.1 ± 0.79. Three patients developed post-operative stiffness of the joint.

Conclusion

Patients with intramedullary screw fixation have good post-operative results with early return to work.

## Introduction

Metacarpal fractures account for 33% of all hand fractures in the United States [1-2]. The most common type of metacarpal fractures are the distal metacarpal fractures. Usually, fractures of the fifth finger also known as the boxer’s fracture are very common. Metacarpal fractures are the second most common type of upper limb fractures, with most common being fractures of the distal radius [3].

Metacarpal fractures are prone to functional deficit when they are inadequately treated. With a little degree of displacement, the treatment can be conservative, but significant displacement requires surgical intervention [4]. The current management includes the use of either Kirschner wires (K-wires) or intramedullary screws. Both of these are well-known methods for the management of metacarpal fractures resulting in little functional deficit. However, intramedullary screw fixation is associated with little or no need for splinting time as compared with the use of K-wires and hence the time period to return back to work is significantly short.

## Materials and methods

**Figure 1:**
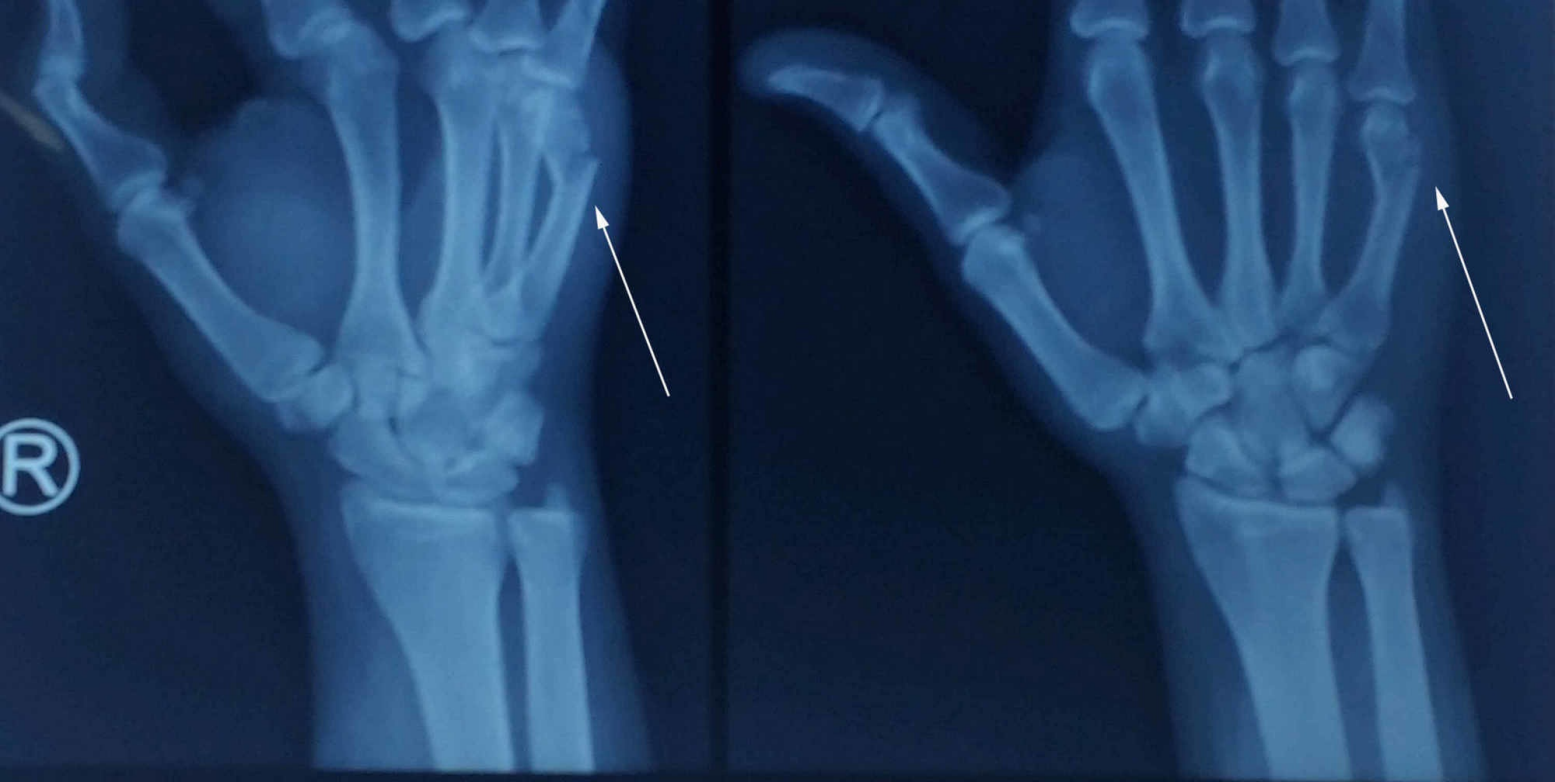
A 43-year-old male patient with metacarpal fracture due to a road traffic accident

This study was conducted at Dr. Ruth Phau Civil Hospital, Karachi (CHK), Pakistan. CHK is a tertiary care teaching hospital located in Karachi and receives the bulk of cases from all over Pakistan. This is a cross-sectional study. The study duration was between August 1, 2018 and January 31, 2019. Inclusion criteria had patients with metacarpal fractures between the ages of 17-50 years. Patients presenting under six hours of injury were included. Patients with extra-articular closed fractures requiring surgical intervention were included. Minimally displaced fractures which could be managed conservatively were not included in the study. Exclusion criteria had patients with intra-articular fractures, open fractures, skeletally immature patients, patients with diabetes mellitus, re-fracture cases, and cases with fracture on the hand which was previously operated. A total of 32 patients meeting the criteria and presenting with metacarpal fractures were included in the study (Figure 1). The pain was analyzed using visual analogue scale (VAS) at the time of presentation. They were managed with 2.4 or 3.0 mm as per templating partially threaded intramedullary headless screw fixation with the length of the screw varying from 30 mm to 50 mm.

**Figure 2:**
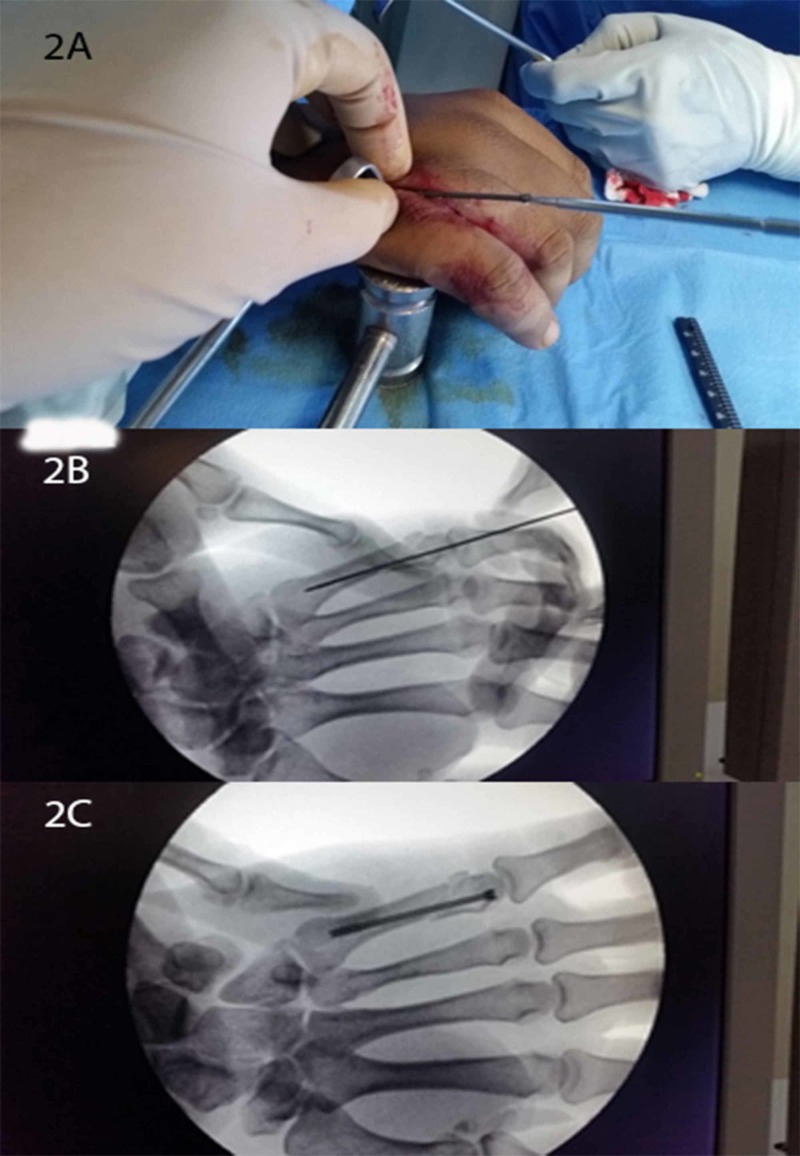
Per-operative pictures of headless screw fixation with a live view (2A) and fluoroscopic views (2B, 2C)

All procedures were performed under general anesthesia. The senior surgeon per-operatively determined the diameter of the medullary canal and analyzed the diameter of the screw to be placed. The fracture was reduced and the guide wire was placed, after which, a partially threaded headless screw of appropriate size was inserted. It was confirmed that the head of the screw was buried under the articular cartilage. Extensor mechanism was repaired. Patients were immobilized with splints for one week (Figures 2A-2C).

**Figure 3:**
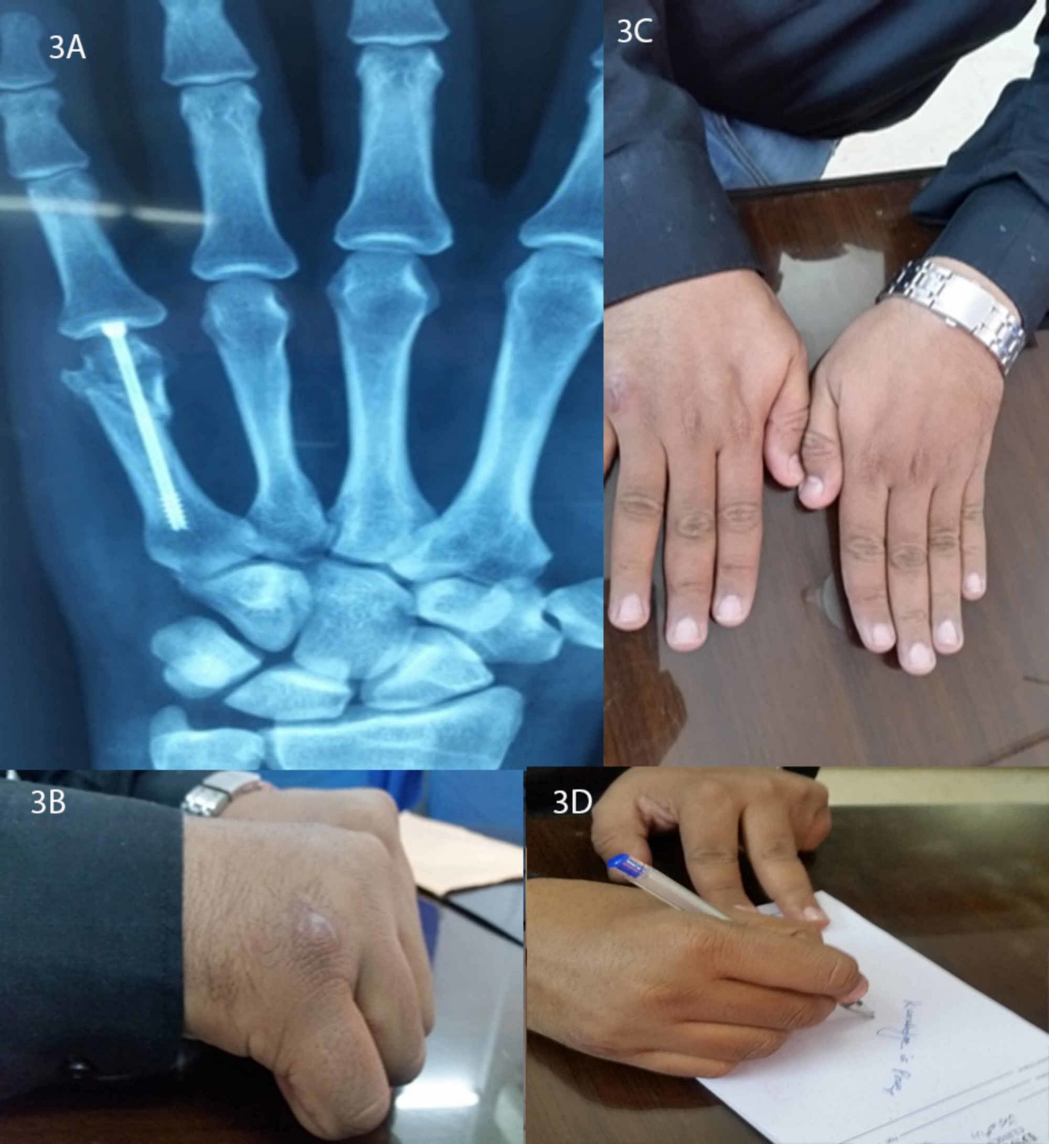
Follow-up presentation of one of the patients with a radiographic presentation (3A) and live views (3B-3D)

Patients were followed post-operatively up to three months with the first visit at one week; later visits were planned at four weeks, eight weeks, and twelve weeks. Activity was commenced one week after surgery. Factors assessed at every follow-up visit include pain using VAS, strength assessment using dynamometer, active range of motion using goniometer, and any disability noticed during routine activities (Figure 3). Patients were asked to rate their level of satisfaction two months after surgery with 1 being least satisfied and 10 being highly satisfied. Their scores are reported in the results.

## Results

Out of the 32 patients with metacarpal fracture, 26 were males whereas six were females. The mean age of patients was 30.9 years ± 13.1 years (range = 17-68 years). The most common cause was road traffic accidents while the second most common cause was falls. In 27 cases, the site of fracture was the distal third of metacarpal bone, three of them had fractures of base of metacarpals and two patients had multiple metacarpal and phalangeal fractures. The mean VAS score for pain at the time of presentation was 7.5 ± 1.7. Intramedullary headless screw fixation was done and patients were followed three months for evaluation. All fractures were united in the mean duration of 46 ± 7 days. Mean post-operative grip strength was found to be 37.8 ± 7.3 kilograms. Mean post-operative total active range of motion in the injured hand was found to be 242.8 ± 14.5 degrees. The average number of days to return back to work were 25 ± 5.4 days. The average patient satisfaction score was 8.1. This is shown in detail in Table 1. Three patients developed post-operative stiffness of joint. Out of these patients, two had multiple metacarpal fractures.

**Table 1:** Demographic variables of patients along with the surgical outcome

Serial number	Age of patient	Gender	Metacarpal fractured	Post-operative grip in injured hand	Post-operative grip in contralateral hand	Total active range of motion in injured hand	Total active range of motion in contralateral hand	Return to work in days	Patient satisfaction score out of 10 (1 is least satisfied and 10 is highly satisfied)
1	22	Male	3	40	45	230	250	20	8
2	27	Male	4	40	47	265	270	23	9
3	68	Female	4	20	25	260	270	25	7
4	50	Male	4	45	47	230	250	20	8
5	19	Male	5	40	48	235	260	30	9.5
6	17	Male	2,4,5	44	50	235	260	35	7
7	30	Male	5	42	50	260	270	20	8
8	32	Male	5	40	45	236	260	15	9
9	19	Male	5	45	50	230	250	30	7.5
10	22	Male	5	37	45	230	250	35	8
11	17	Male	5	40	45	230	260	23	8.5
12	18	Male	3	38	40	260	260	22	8
13	23	Female	3	25	27	237	250	20	8
14	27	Male	3	42	45	250	260	25	8
15	29	Male	4	44	40	233	270	26	8
16	33	Female	3	20	22	235	260	22	7
17	30	Female	5	25	28	250	270	30	9
18	55	Male	4	40	45	244	260	31	8.5
19	47	Male	5	35	43	240	260	34	8
20	57	Male	4	37	45	240	250	18	8
21	20	Male	3	42	45	245	250	19	9
22	21	Male	3	40	45	250	250	22	7
23	22	Male	5	35	43	270	270	24	8
24	23	Male	5	47	50	225	250	26	9
25	24	Male	4	42	50	235	250	27	7
26	25	Male	3	42	50	225	240	26	9.5
27	33	Male	2	40	50	235	250	22	8
28	43	Male	5	35	45	270	270	19	9
29	32	Female	4,5	22	25	220	250	34	7
30	19	Male	3	42	45	260	250	22	9.5
31	40	Male	2	43	45	235	250	33	8
32	45	Female	5	42	47	270	270	25	9

## Discussion

There are other studies which have compared the results of intramedullary fixation with the use of K-wires and supported the use of intramedullary screws [5]. Boulton et al. reported full extension and 80° interphalangeal joint flexion after intramedullary screw fixation of metacarpal joint of their patient [6]. Ruchelman et al. report their study results which were conducted on 39 patients with intramedullary joint fixation. The mean grip strength of fractured hand compared to the other hand was 105% whereas the mean metacarpopharangeal joint flexion was 88°. The post-operative flexion-extension arc for their group of patients was 90° [7]. Coucerio et al. compared the use of K-wires and intramedullary screw fixation among patients with fractures of equal severity in both groups. They reported no significant difference in pain and strength of degree of mobility among both groups; however, the post-operative course was significantly short in patients managed with intramedullary screws and they could return back to work early [5]. Beck et al., in their literature review, compared the results of five articles and concluded that intramedullary headless screw fixation has benefits over K-wires as they are associated with early mobility and also save patients from the removal of K-wires [1]. Tobert et al. report the results of their retrospective study. They report results of intramedullary headless screw fixation of 18 metacarpal fractures; there were no post-operative complications among patients whereas active motion was possible after one week of the surgery [8].

Our study reports post-surgical mean grip strength of 37.8 kgs and the mean total active range of motion of 242 degrees which proves that intramedullary headless screw fixation is a good surgical management option associated with an early post-operative course. The average number of days to return back to work for the patients of our study was 25. The other surgical modality, the use of K-wires is associated with post-operative complications. The complication rate of K-wire for fixation can exceed up to 16%. These complications may include non-union, infection leading to osteomyelitis, and pin-pull out [9]. Jann et al. reported results of 20 unstable metacarpal fractures which were surgically managed with compression screws. Out of 15 patients, one required arthrolysis and one had extension lag. No other complications were observed. The post-operative healing time reported for these patients was six weeks without the need of removal of screws [10]. Raghavendra et al. conducted a study which involves the management of metacarpal fractures with either K-wires or headless compression screw. The most common complication in their study was finger stiffness. They reported less post-operative course with the use of screws [11].

Avery et al. conducted a study comparing the biomechanical stability of K-wire fixation to intramedullary headless screw fixation. They concluded that the latter is associated with greater strength resulting in less failure and bending [12]. However, Melamed et al. conducted a study that compared the post-operative biomechanical differences of unstable metacarpal fractures treated with either intramedullary headless screw or dorsal plates. They concluded that dorsal plate fixation is a more reliable method of joint fixation as compared to intramedullary headless screws and offers more mechanical stability [13].

The limitation of this study includes a small sample size. Further studies comparing the use of K-wires vs. intramedullary headless screw fixation should be done among patients with a similar degree of fractures.

## Conclusions

In conclusion, patients with intramedullary screw fixation have good post-operative results with early return to work. Patient satisfaction was also good. This can be considered as a better surgical management option as compared to K-wire fixation.
